# The Physical Factors in Photo-Therapy

**Published:** 1903-11-21

**Authors:** 


### THE PHYSICAL FACTORS IN PHOTO-THERAPY.

Some interesting experiments have been carried out by J. E. Barnard and H. de R. Morgan[1] with the object of ascertaining to what agencies may be attributed any therapeutic effects that the light treatment may produce. The first question that requires solution is whether the effect of the light treatment on any specific disease, such as lupus, is due to bactericidal power of the light or to the reaction which it excites in the tissues themselves. This question they have as yet been unable to decide. Although it is well known that light, without heat, destroys micro-organisms outside the body, it may be doubted if a similar result will be produced on bacteria lying in the tissues of the body. They point out that light is powerless to destroy bacteria in those cases where its rays are made to pass through any organic substance before impinging on the bacteria, even the thinnest film of agar, for instance, serving as a protection. Much less can the bactericidal rays penetrate living or dead tissue under the ordinary conditions of experiment. This was proved by the following experiment. The light from an arc lamp was allowed to fall on part of the surface of an agar plate which had been thickly inoculated with an active culture of bacillus coli communis. In order to exclude heat the rays from the arc lamp were made to pass through a cylinder through which water was kept circulating. Even after so short an exposure as eleven seconds the comparative number of surface colonies was greatly reduced in that part of the plate which had been exposed to the light, but those in the unexposed portion of the plate and those colonies in the depth of the agar were unaffected, even after two hours' exposure. A somewhat similar experiment showed that a portion of human skin was as efficient a protector of the colonies as was a film of agar, and the same was true both of a living and a dead frog's foot. A very pretty illustration is shown of an agar plate subjected to the latter experiment, in which the surface bacilli have been destroyed around the frog's foot, while those which lay under the foot and so were protected from the light have grown, and the shape of the foot is clearly marked out by the growth. From these experiments it is concluded that, the bactericidal rays being non-penetrative, the therapeutic effects of light may be due to the reaction produced in the tissues. They then proceeded to try and differentiate the bactericidal rays from those which excite a reaction in the living tissues. Although the bactericidal rays could be exactly localised in the spectrum, those which are capable of setting up a tissue reaction could not be so definitely located. The bactericidal rays were found to occupy the middle third of the ultra-violet portion of the spectrum, and were localised by exposing culture plates to the spectrum of an arc lamp. The portions of the culture plate on which the visible spectrum fell were unaffected, and the same was true for a short space beyond the visible violet of the spectrum, then there came an area corresponding to the middle third of the ultra-violet portion of the spectrum where growth was prevented. Although the rays causing tissue reaction could not be exactly located in the spectrum, it was shown that they belonged to the ultra-violet portion by the fact that they would not pass through glass. An important fact was discovered concerning the bactericidal rays, namely, that the interposition of water considerably diminished their power of destroying micro-organisms. Thus it was found that an exposure of five minutes without the water-circulating apparatus had a greater germicidal effect than a 25-minute exposure with it. In other words, the light on passing through 2·5 cm. of water lost four-fifths of its bactericidal power. They therefore conclude that in photo-therapeutics the generally-used water-cooling apparatus might be dispensed with if the heat could be eliminated by other means, assuming that the directly bactericidal rays are the essential ones, which at present is by no means certain.

[1] British Medical Journal, November 14.

